# Genomic Regions, Molecular Markers, and Flanking Genes of Metribuzin Tolerance in Wheat (*Triticum aestivum* L.)

**DOI:** 10.3389/fpls.2022.842191

**Published:** 2022-05-19

**Authors:** Benjamin Kurya, Md Sultan Mia, Hui Liu, Guijun Yan

**Affiliations:** ^1^UWA School of Agriculture and Environment, The University of Western Australia, Perth, WA, Australia; ^2^The UWA Institute of Agriculture, The University of Western Australia, Perth, WA, Australia; ^3^Department of Primary Industries and Regional Development (DPIRD), South Perth, WA, Australia

**Keywords:** single nucleotide polymorphism (SNP), genome-wide association studies (GWAS), quantitative trait loci (QTL), chlorophyll content index (CCI), marker-assisted selection (MAS), herbicide tolerance, PSII, ROS

## Abstract

Understanding the genetics of metribuzin (a group C herbicide) tolerance in wheat is vital in developing tolerant cultivars to improve wheat productivity in dryland farming systems. This study investigated metribuzin tolerance in wheat by conducting a Genome-wide Association Studies (GWAS) with a panel of 150 wheat genotypes of diverse genetic backgrounds and genotyped them with the wheat 90 K SNP genotyping assay. The phenotyping was conducted in a temperature-controlled glasshouse at the University of Western Australia (UWA). Genotypes were sprayed with a metribuzin dose of 400 grams of active ingredient (g. a.i.) ha^−1^ as pre-emergent in a specialized spraying cabinet and transferred to the glasshouse where the tolerance level of the genotypes was assessed by measuring the relative reduction in chlorophyll content of the leaves. The decrease in chlorophyll content of the treated plants compared to the control was regarded as the phytotoxic effects of metribuzin. GWAS analysis following a mixed linear model revealed 19 genomic regions with significant marker-trait associations (MTAs), including ten on chromosome 6A, three on chromosome 2B, and one on chromosomes 3A, 5B, 6B 6D, 7A, and 7B, respectively. Sequences of the significant markers were blasted against the wheat genome, IWGSC RefSeq V1.0, and candidate genes having annotations related to herbicide tolerance in wheat, especially in pathways reported to be involved in metribuzin tolerance, such as cytochrome P450 pathways and ATP Binding Cassette (ABC) superfamilies, were identified in these genomic regions. These included *TraesCS6A01G028800, TraesCS6A02G353700, TraesCS6A01G326200, TraesCS7A02G331000, and TraesCS2B01G465200.* These genomic regions were validated on 30 top tolerant and 30 most susceptible genotypes using the five closest SSR makers to the flanked SNPs. Sufficient polymorphism was detected on two markers (*wms193* and *barc1036*) that were found to differentiate between the susceptible and tolerant alleles and a t-test analysis of the phenotypic data shows a significant (value of *p* < 0.001) difference suggesting that these markers can be used for marker-assisted selection (MAS) in metribuzin studies and wheat breeding programs.

## Introduction

Wheat is a valuable cereal crop globally, supplying four billion people with a daily portion of calorie intake (Liu et al., 2019; Venske et al., 2019). In Australia, wheat is a major grain crop, contributing about 12% to global trade (Kleemann and Gill, 2008). Due to the wide adoption of no-till or zero tillage cropping systems in Australia, a major constraint for wheat production is weed infestation which can cause yield loss of up to 50% (Kleemann and Gill, 2008). High weed infestation during the early seedling stage hinders effective tillering in wheat leading to poor yield (Pilcher et al., 2017; Bhoite et al., 2018). Hence, controlling weeds is a critical aspect in sustaining wheat production. A cheap, effective, and convenient method of weed control for agricultural production is the use of herbicides. It is vital that such herbicides are tailored to favor crops and destroy weeds to avoid crop damage (Ponciano, 2018). However, there are genotypic variations in plants for herbicide tolerance, and in particular conditions, herbicides may cause toxicity to plants, especially in susceptible genotypes.

Metribuzin is a broad-spectrum triazine herbicide used in wheat fields for weed control. It is classified under group C, known under the IUPAC name 4-Amino-6-(1,1-dimethylethyl)-3-methylthio-1,2,4-triazine-5(4H)-one (Bhoite et al., 2018). When metribuzin is applied to plants, it is primarily absorbed by the roots *via* diffusion and translocated to the shoots. The site of action (SOA) of metribuzin in the photosystem II protein complex (Beckie et al., 2019). The absorbed metribuzin inhibits photosynthesis by binding to the D1 quinone protein of the photosystem II (PSII) in the chloroplast and prevents electron transport that is necessary for the conversion of light to energy (Si et al., 2006; Pilcher et al., 2017; Bhoite et al., 2018; Ponciano, 2018). This chain reaction leads to lipid peroxidation, which causes degradation of chlorophyll, carotenoids and cell membrane, and eventual tissue death (Javid et al., 2017; Bhoite et al., 2018; Ponciano, 2018). Metribuzin is commonly used as a pre- and post-emergence herbicide for the control of annual grasses and broad leaf weeds such as bluegrass (*Poa annua*), corn buttercup (*Ranunculus arvensis*), brome grass (*Bromus diandrus*), barley grass (*Hordeum glaucum and H. leporinum*), and Italian ryegrass (*Lolium perenne* spp. *multiflorum*) in different farming systems including dryland systems such as the wheat cropping areas of Western Australia (Pilcher et al., 2017; Bhoite et al., 2018; Ponciano, 2018). Most wheat cultivars tolerate metribuzin at the recommended dose rate, i.e., 225–300 ml/h^−1^ (Pilcher et al., 2017). However, some cultivars are highly susceptible to metribuzin leading to crop damage and ultimately poor yield (Villarroya and Garcia-Baudin, 2000; Bhoite et al., 2019). Hence, the identification of tolerant sources can facilitate the development of wheat cultivars tolerant to metribuzin.

Many successful investigations in the past have identified wheat cultivars tolerant to metribuzin. For example, Pilcher et al. (2017) reported the outcome of a screening investigation of 86 genotypes and found nine to be highly tolerant. Similarly, Kleemann and Gill (2007) identified four wheat cultivars that showed sufficient tolerance to metribuzin. Bhoite et al. (2017) screened 946 genotypes and identified nine to be highly tolerant to metribuzin application. Identification of metribuzin tolerant cultivars through screening involves testing different dose rates and field screening which can be expensive and time-consuming. Understanding the genetic basis of wheat tolerance to metribuzin could lead to the development of molecular markers with the potential to accelerate genetic gain in breeding programs by at least partially reducing the number of herbicide trials. However, unlike studies on metribuzin sensitivity in wheat, the genetic basis of metribuzin tolerance has received much less attention. Investigations by various authors claimed that the mechanism controlling metribuzin tolerance in wheat is complex and poorly understood because the complexity of herbicide tolerance involves the interaction of different mechanisms relating to the site of action (SOA) and metabolic detoxification (Bhoite et al., 2018).

Different studies have reported the mode of inheritance of metribuzin in soybean and potato controlled by a single recessive gene (Runyan et al., 1982; Fischer, 1983; Ratliff et al., 1991; Villarroya and Garcia-Baudin, 2000; Bhoite et al., 2018). No conclusive outcomes have been reported in wheat. Villarroya and Garcia-Baudin (2000) reported that both nuclear and cytoplasmic inheritance might control metribuzin inheritance in wheat. Bhoite et al. (2019) confirmed that metribuzin tolerance in wheat is additive and identified potential candidate genes for metribuzin tolerance at the seedling stage in wheat based on tolerance segregation in a bi-parental mapping population derived from crosses between a susceptible and tolerant wheat cultivar. The study by Bhoite et al. (2019) sets the foundation for identifying QTLs associated with metribuzin tolerance in wheat, yet it is based on a narrow genetic diversity. However, an investigation focusing on a densely genotyped germplasm set with a wider range of natural variation arising from years of recombination events and fixed alleles would offer a substantial opportunity to develop markers for marker-assisted selection (MAS). In all the previous Genome-wide Association Studies of metribuzin tolerance in wheat (as in Pilcher et al., 2017; Bhoite et al., 2019), metribuzin was used as post-emergent. However, in Australia metribuzin is mainly used as pre-emergent in wheat and so far, no association study has been reported where metribuzin was used as pre-emergent.

GWAS is a well-established approach for the marker-trait association to detect genetic variations associated with a complex trait (phenotype) in an unstructured population (Huang and Han, 2014; Jia et al., 2018; Luo et al., 2019; Alqudah et al., 2020). GWAS was initially introduced for human genomic research about a decade ago (Huang and Han, 2014; Scherer and Christensen, 2016). In the past few years, the introduction and application of GWAS in plants have led to major successes in crop improvement (Luo et al., 2019). The GWAS approach has been used for marker-trait association in many field crops such as maize, rice, sorghum, and soybean (Huang and Han, 2014; Luo et al., 2019), *Brassica* spp. (Gacek et al., 2017), barley (Alqudah et al., 2020), and wheat (Battenfield et al., 2018). GWAS analysis can be considered as conducting multiple regression analysis between the phenotypic data and the SNP markers to find associations (Burghardt et al., 2017). Therefore, this current investigation aims to use 150 wheat cultivars from diverse backgrounds to perform a GWAS using the wheat 90 K Illumina iSelect genotyping assay to identify and validate genomic regions associated with metribuzin tolerance in wheat.

## Materials and Methods

### Genotypes and Their Backgrounds

Seeds of 150 wheat genotypes used for this study were sourced from the germplasm collection of the Australian Grains Gene (AGG) bank, wheat breeding companies/institutions including InterGrain Pty Ltd., Australian Grain Technologies, LongReach Plant Breeders, Edstar Genetics Pty Ltd., Inner Mongolia Academy of Agricultural and Animal Husbandry Sciences, and Gansu Academy of Agricultural Sciences. This panel of 150 accessions originates from 20 different countries across 6 continents of the world. The origin, genus, and species are presented in Supplementary Table S1. Many accessions used for this investigation are among the 946 accessions that were characterized by Bhoite et al. (2017) for metribuzin studies that reported a wide range of variability and response to metribuzin application.

### Experimental Design and Treatments

**Figure 1:**
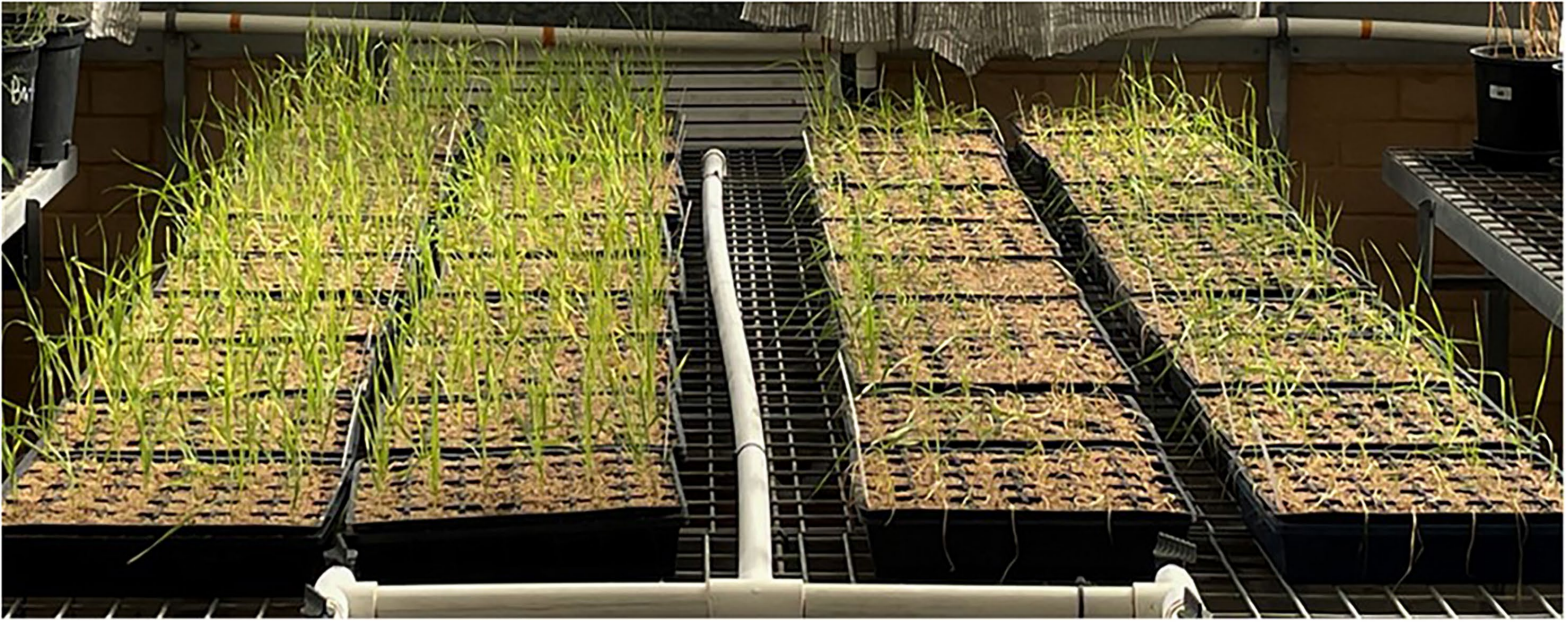
Phenotypic screening of 150 wheat genotypes response to metribuzin application showing treated plot (right) and control plot (left).

The experiment was carried out in a well-equipped temperature-controlled glasshouse at the University of Western Australia (31°57′S, 115°47′E). The experiment was laid out in a randomized complete block design (RCBD) and replicated four times in both treated and control plots. K30-kwikpot seedling trays were used to screen both treated and control plots. The seedling trays consisted of 5 × 6 uniform cells (30 cell/tray), such that each cell represented one genotype, and five trays accommodated 150 genotypes. The seedling trays were filled with homogenous river sand on a glasshouse bench and watered to 100% field capacity. After 48 h, single seeds were sown at 1.5 cm depth in the middle of each cell. The treated plots (20 trays) were sprayed with a metribuzin dose of 400 g a.i. h^−1^ in a cabinet spray chamber with a flow rate of 106.9 l h^−1^ following the procedure described in Bhoite et al. (2018). The trays were returned to the glasshouse and randomized on the bench; watering was suspended for 24 h. The control plots were sprayed with only water and separated from the treated plot but kept on the opposite side of the bench while being monitored together (Figure 1).

### Phenotypic Data Collection and Analysis

Data collection started when two fully expanded leaves (Zadok’s scale 12) were observed on the germinated seedling, and visible symptoms of phytotoxicity were noticed in some of the plants (Zadoks et al., 1974). Herbicide damage was assessed by measuring the relative chlorophyll content of the leaves using a hand-held Minolta SPAD-502 chlorophyll meter (Spectrum Technologies Inc. Plainfield, IL, United States) from a fully expanded leaf. A chlorophyll content Index (CCI), measured as the per cent reduction in chlorophyll content of the treated plants compared to the control plants, was regarded as the effects of metribuzin. Hence, lower CCI indicated higher tolerance and vice versa. Five genotypes failed to germinate during the experiment, and the final genotypes analyzed at the end of the experiment were 145. The CCI was calculated from the formulae:


$$\mathrm{CCI}=\frac{\begin{array}{c} \mathrm{chlorophyll content}\;\left(\mathrm{CC}\right)\mathrm{of the control plant}\\ -\mathrm{CC}\;\mathrm{of the treated plant}\; \end{array}}{\mathrm{CC}\;\mathrm{of the control plant}}\times 100$$

Phonotypic data were subjected to a group variance test using the Breusch Pagan test with a null assumption of equal group variance. This was followed by a Shapiro test of normality with the null assumption that data are normally distributed. To understand the variation in metribuzin effect among the accessions, a one-way ANOVA was performed in R software by following a fixed effect model: Y ij = μ + g j + ε ij, where Y ij is observed mean, μ population mean, gj effect due to the jth genotype, and ε ij is random error. The broad sense heritability (H2) of the panel was computed based on the ratio of the phenotypic variance to the genotypic variance using the following formula: H^2^ = δ^2^g/(δ^2^g + δ^2^e), where δ^2^g and δ^2^e are the estimated genotypic and error variances, respectively. The estimated genotypic and error variances were calculated as δ^2^g = (MSg − MSe)/r and δ^2^e = (MSg − MSe)/r, where MSg = mean square of the accessions, MSe = the residual error, and *r* = the number of replicates.

### Wheat 90 K SNP Illumina iSelect Assay Genotyping

DNA samples were extracted from individual genotypes on 3-week-old plants using the cetyl trimethyl ammonium bromide (CTAB) method and stored in TE buffer (pH 8.0; Sharp et al., 1988). High-quality DNA was ensured from the agarose gel electrophoresis and NanoDrop 2000C. A Qubit fluorometer (2.0) was used to assess the DNA concentration utilizing the Qubit ds DNA broad range assay. The populations were genotyped using the wheat 90 K Illumina iSelect array and analyzed by genome studio v2.0 software (Illumina Inc.), following the protocol described previously by Wang et al. (2014). Quality control for genotype calling was done by either removing SNP markers with less than 80% call frequency or excluding SNPs with minor allele frequency (MAF) ≤ 5%. SNPs with > 0.25 heterozygous calls were also removed.

### Population Structure and Linkage Disequilibrium (LD) Analysis of the Panel

The population structure of the studied materials was determined following a Bayesian clustering model in STRUCTURE V2.3.4 using K (number of subpopulations) values ranging from 2 to 9. For each K value, five independent runs were performed based on an admixture model, where each run was carried out with 10,000 recorded MCMC (Markov-Chain Monte Carlo) iterations and 10,000 burn-in periods. The output was visualized in Structure Harvester software, and the optimal K number was determined using the second-order change rate of the probability function with respect to K (ΔK). NJ trees and kinship matrix were produced with the software Tassel v5.0 and investigated to validate population stratification.

Pairwise LD among the markers was calculated as a squared allele frequency correlation (*r*^2^) between SNP marker pairs in Tassel 5.2.71 with a sliding window of 50 markers. Using a custom R script, the obtained *r*^2^ values were plotted against the physical position of the SNPs obtained from the IWGSC RefSeq v2.0 reference genome. A locally weighted polynomial regression (LOWESS) curve was fitted to display the LD decay. LD decay rate was measured as the physical distance at which the average pairwise *r*^2^ dropped to half of its maximum value. LD decay was calculated for the whole genome, the three sub-genomes, and individual chromosomes.

### GWAS Analysis

GWAS, a marker-trait association tool, was used to map associations between phenotypic traits (CCI) and SNP markers using Tassel v5 (Bradbury et al., 2007). GWAS analysis was carried out using a mixed linear model (MLM) and a kinship matrix using five principal components and Bonferroni as correction (*α* = 0.05; Scherer and Christensen, 2016; Mourad et al., 2018; Xu et al., 2020). The first five principal components accounted for 26.5% of the total variance among the studied materials (Supplementary Table S2; Supplementary Figure S1). The optimal number of PC was determined by investigating respective contribution, elbow point of scree plot, and distribution pattern in the Q-Q plot. The analysis was initially carried out with a general linear model (GLM): Q matrix (Q + PCA), which generated over 100 associations, and then finally with a mixed linear model (MLM): kingship matrix (K, PCA + K; Q + K) to control for any false associations and population structure due to multiple levels of relatedness (Xu et al., 2020). Hence, MLM generated fewer and stronger associations. To reduce the Type II error rate, a threshold of -log10 (value of *p*) > 4 was set to call significant QTL (Voss-Fels et al., 2017). GWAS analysis output from TASSEL was then used to prepare Manhattan and Q-Q plots using the R package rMVP on R 4.0 (Yin et al., 2021).

### Identification of Candidate Genes

Significant SNP makers identified in the initial analysis were then searched in the Grains Genes database,^1^ where the physical position of markers was identified within the wheat genome IWGSC RefSeq V1.0. Genes spanning the SNPs genomic region or within 2 Mbp (upstream or downstream) of flanked SNPs markers were analyzed for annotations related to herbicides tolerance, as Brodie et al. (2016) theorized that SNPs might be up to 2 Mbp away from their associated genes. The *Traes* IDs of the putative genes acquired from the reference genome were further searched on the Ensemble database^2^ to identify gene function utilizing the high confidence gene annotation of wheat reference genome IWGSC RefSeq V1.0.^3^ Additional information on molecular and biological functions of the identified genes was also investigated on the InterPro website.^4^ SNPs that were associated with herbicide tolerance or stress response in plants were considered as putative candidate genes controlling metribuzin tolerance in wheat.

### Marker Validation

To validate the genomic regions identified through the GWAS, 30 most tolerant genotypes and 30 most susceptible genotypes identified from the experiment were selected, and fresh leaf samples of the individual genotype were collected for DNA extraction. A cetyl trimethyl ammonium bromide (CTAB) method was used for DNA extraction and then suspended in a 50 μl of TE buffer. A 0.2 μm (0.6 μl) mix of forward and reverse primer (primers were sourced from Sigma Aldrich Oceania Pty Ltd. NSW, Australia) was added to 5 μl Takara EmeraldAmp max master mix containing Taq, MgCl_2_, dNTPs, and a final 1 μl of DNA sample making a total volume of 15 μl. The PCR reaction was performed using an Eppendorf master cycler programmed at 98^o^ C for 2 min, in 30 cycles at 98^o^ C for 1 min and 50°C annealing temperature for 30 s, 72^o^ C for 30 s, and a final extension at 72°C at 7 min (Xu et al., 2020). A 2% agarose and TBE buffer were used for running electrophoresis for 25 min at 200 volts, and the gel images were visualized using an Eppendorf gel documentation system. The polymorphic bands from gel images for tolerant genotypes were scored as “AA” in the validation process, and the susceptible alleles were scored as “aa” alleles.

## Results

### Phenotypic Response of Wheat Genotypes to Metribuzin Application

**Figure 2:**
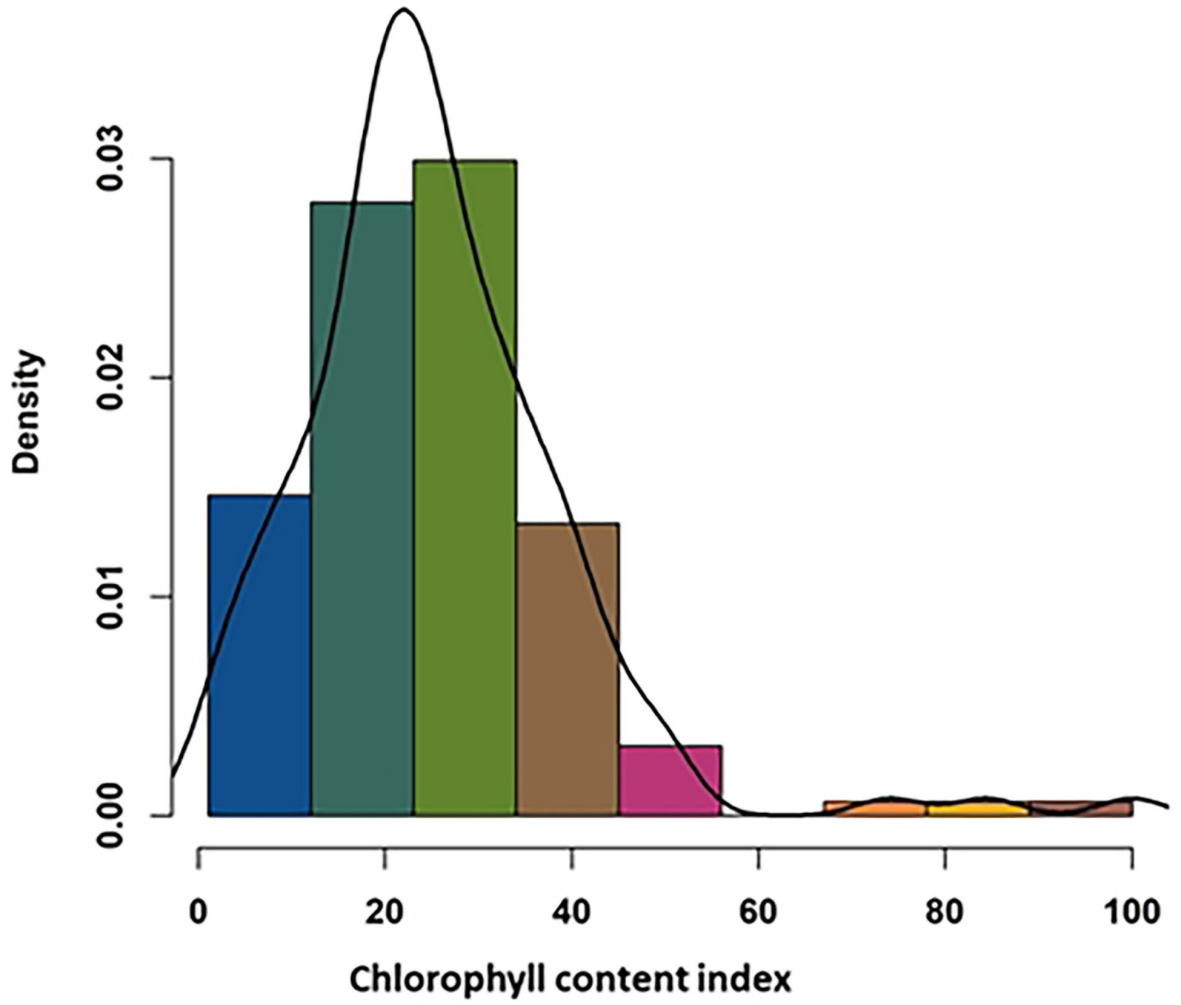
Frequency distribution of 145 wheat genotypes response to metribuzin application based on the assessment of the chlorophyll content index.

SPAD measurement of the leaves showed a wide range of variation in relative chlorophyll content among the studied genotypes (Supplementary Table S1). The minimum leaf chlorophyll content for the control plant was 27.1, and the maximum was 46.2, whereas those for treated plants were 4.8 to 40.9, respectively. Percent reduction in the chlorophyll content in response to the application of metribuzin ranged from 1.1 to 84.5 (Figure 2; Supplementary Table S1). The ANOVA analysis results show a significant difference (*p* < 0.001) in CCI among the 145 genotypes following metribuzin application and indicate that sufficient genetic variation exists among the genotypes that can facilitate a positive outcome through GWAS. The heritability (H2) of CCI measure for the 145 accessions was 0.74 and a genotypic variance of 28.69 (Supplementary Table S3).

### Marker Distribution, Genetic Diversity, and LD Decay of the Wheat Panel

**Figure 3:**
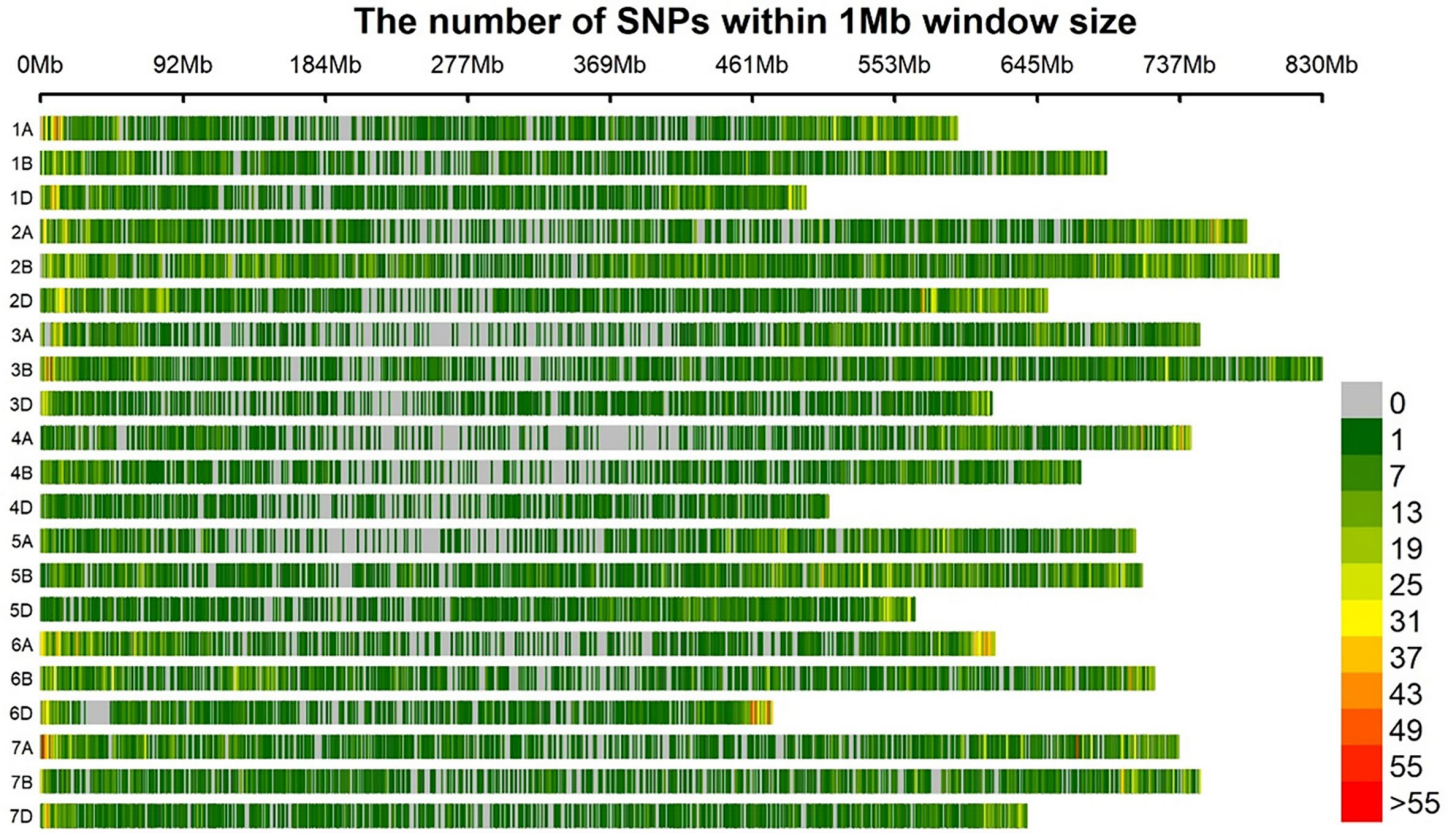
Distribution of SNPs on different chromosomes of the wheat genome.

After filtering, there are a total of 46,287 SNP markers remained that were used for association studies in this current investigation. Figure 3 and Supplementary Table S4 show genome-wide marker number, density, and distribution. The B subgenome has the highest number of SNP markers (18,940 SNPs) with a density of 3.7 SNP markers/MB. This is followed by the A subgenome having a total of 15,509 SNP markers with a marker density of 3.1 SNP/Mb. The subgenome with the least SNP number and density is the D subgenome. SNPs are distributed across the genome, i.e., across all the 21 chromosomes of wheat. The chromosome with the highest marker density is 2B having a density of 5.6 Mb, harboring over 4,000 SNP markers. The chromosome with the least SNPs is 4D having only 817 SNPs and a density of 1.6 SNP/Mb.

**Figure 4:**
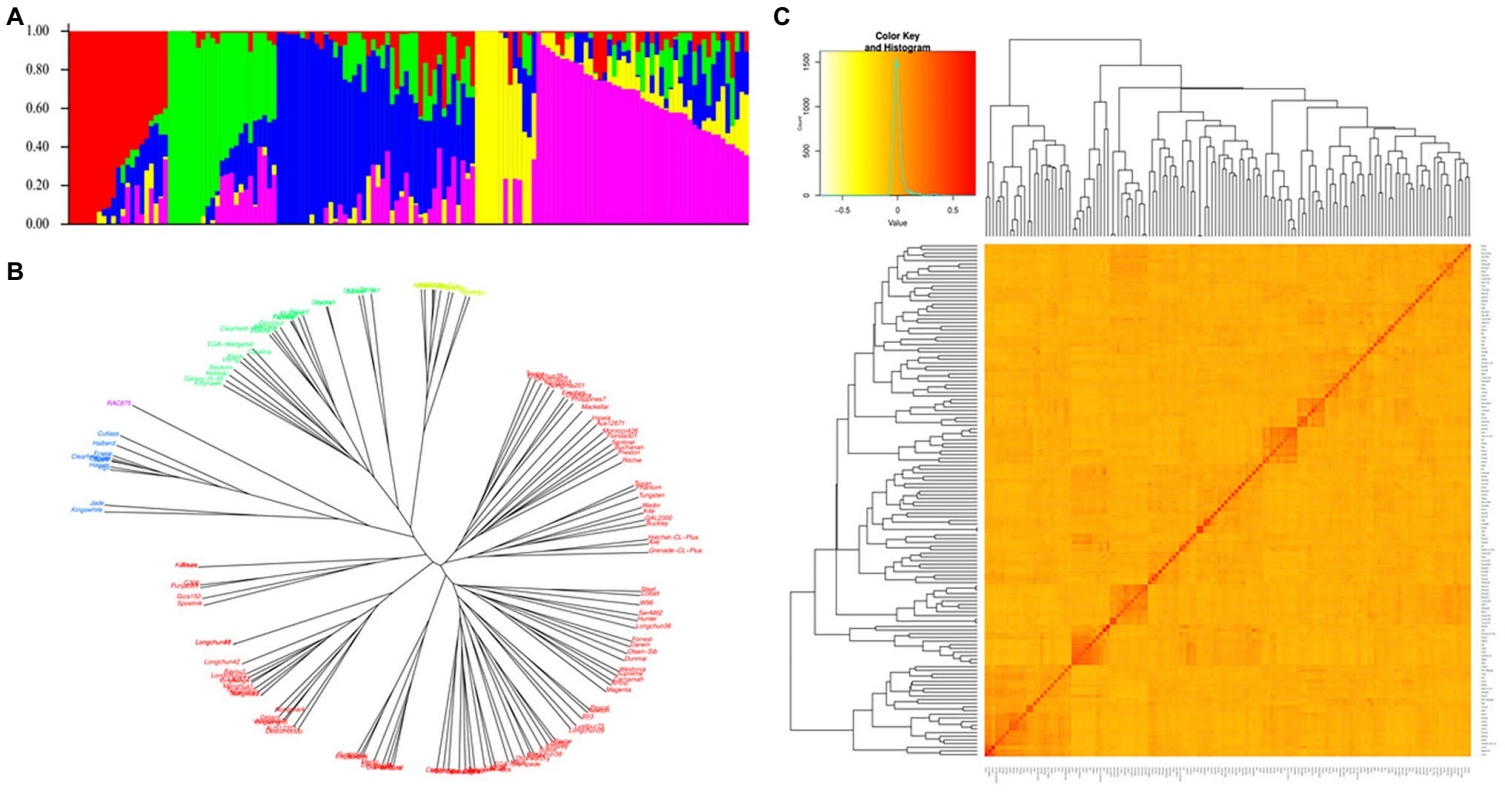
Genetic diversity of the 146 wheat lines. **(A)** Population structure inferred by STRUCTURE at *K* = 5, **(B)** Neighbor-joining tree, and **(C)** Heat map of relatedness (kinship) among the studied materials.

The genetic diversity of the studied materials is shown in Figure 4. The model-based population structure analysis showed that the highest ΔK peak at *K* = 5 indicated the presence of five subpopulations in the panel. The neighbor-joining phylogenetic trees also showed that the wheat lines from different countries were randomly distributed across the five major clusters highlighted in five different colors (red, blue, green, yellow, and violet). The degree of allele sharing among the studied lines has been depicted in the kinship matrix. The matrix showed large proportion of yellow with shades of red in the middle indicating a stratified population structure among the genotypes as represented in other analyses.

**Figure 5:**
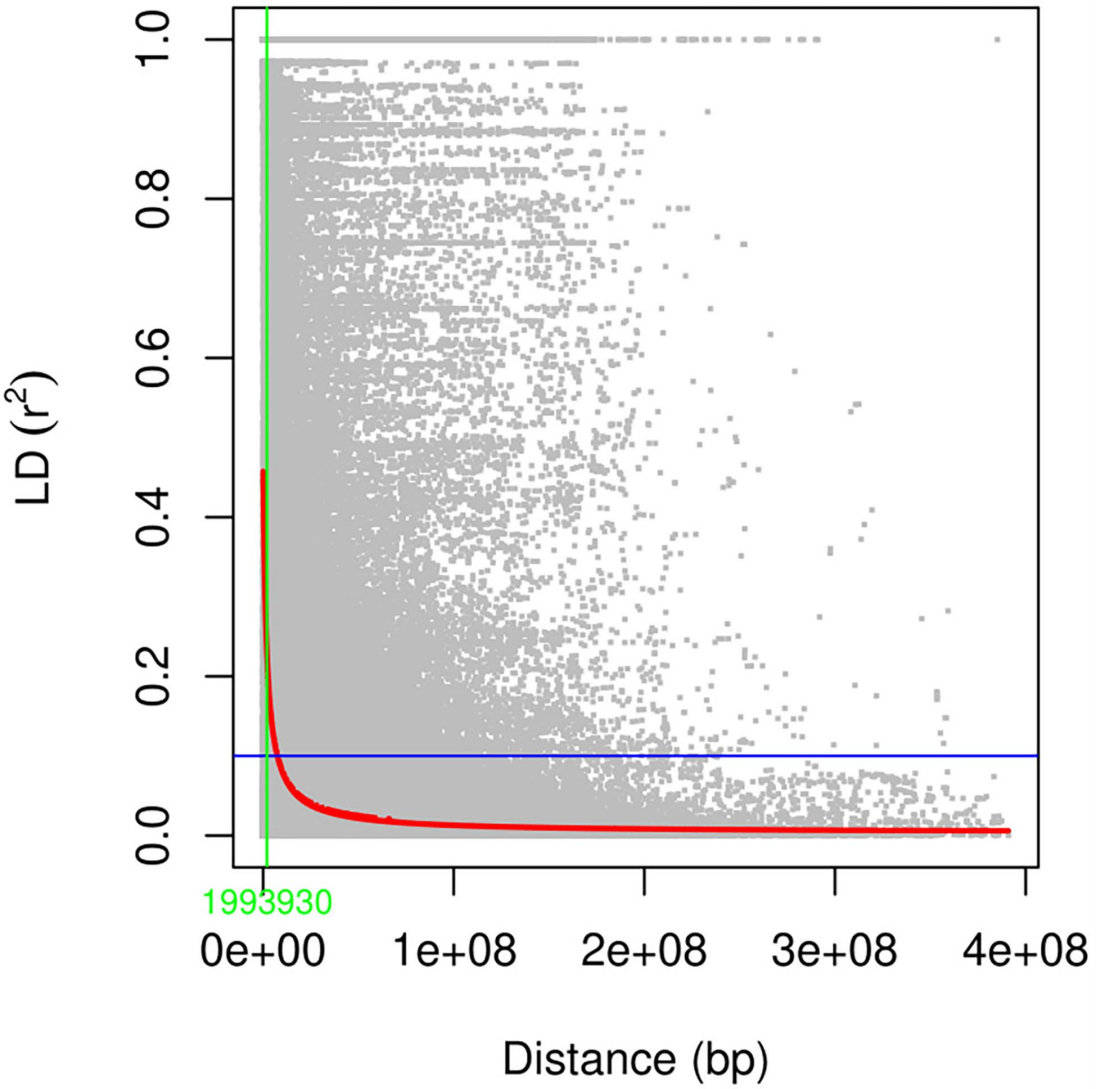
Linkage disequilibrium (*r*^2^) across the whole genome plotted against the physical distance of the SNPs.

The analysis of linkage disequilibrium showed that the LD decayed rapidly with the increasing physical distance. Half decay distance in the studied panel at arbitrary nominal level of *r*^2^ = 0.1 was found to be 1.99 Mbp at the whole-genome level (Figure 5). In the case of sub genome A, B, and D, this value was 1.3, 3.17, and 1.47 Mbp, respectively (Supplementary Table S4). Among the individual chromosome, chromosome 1B had the highest LD decay value (6.79 Mbp), whereas 7D had the lowest (0.45; Supplementary Table S4).

### GWAS Outcomes

**Figure 6:**
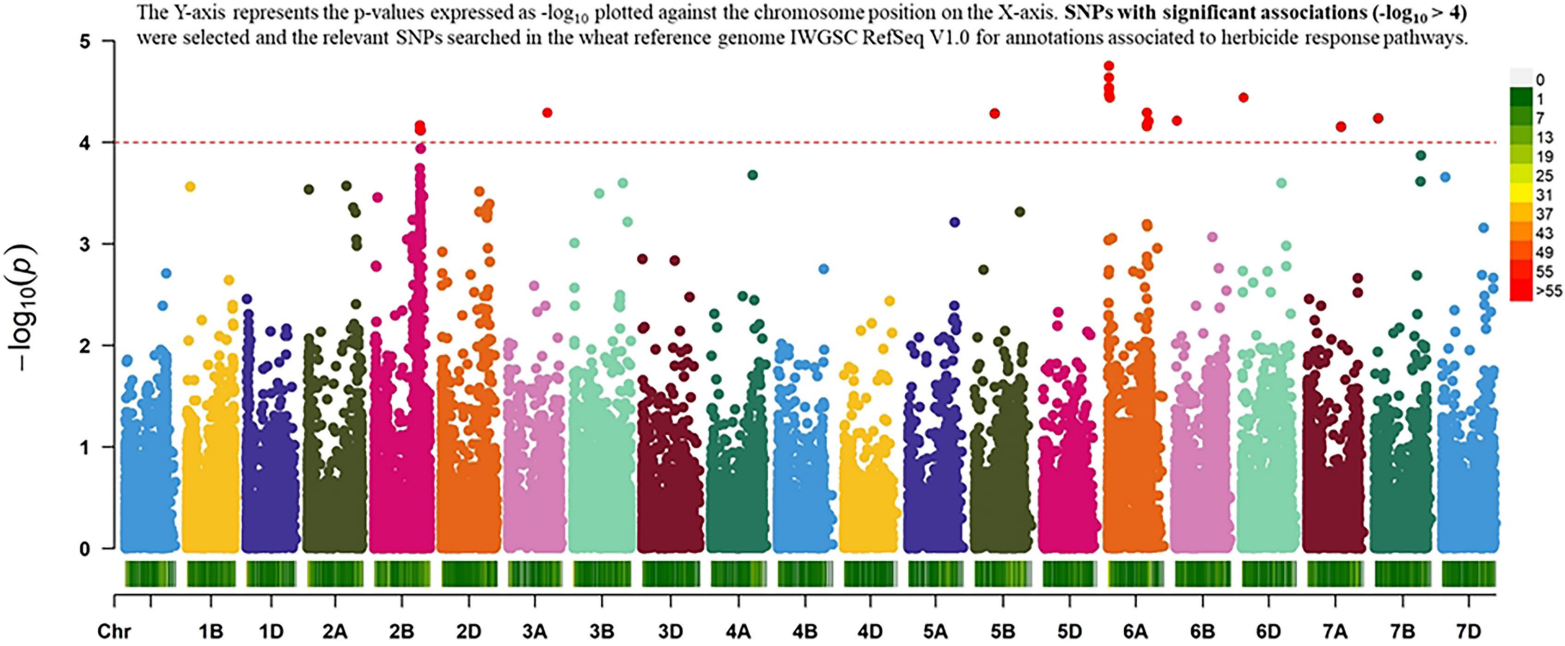
Manhattan plot resulting from GWAS for metribuzin tolerance showing the association between chlorophyll content index and SNP data.

**Figure 7:**
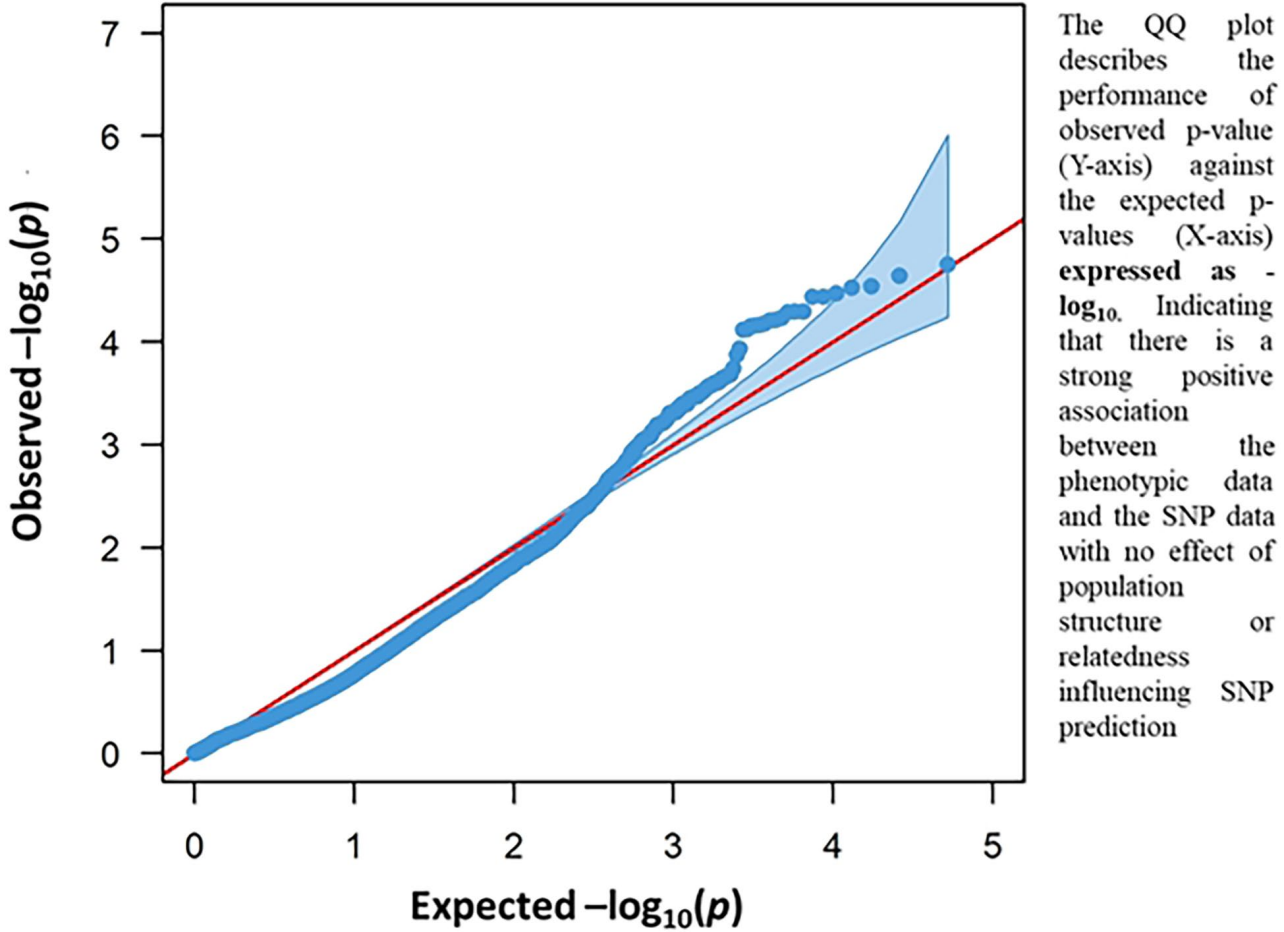
Q-Q plot resulting from GWAS for metribuzin tolerance showing the association between chlorophyll content index and SNP data.

The result of the GWAS analysis is described using a Manhattan plot showing the distribution of SNPs across the wheat genome (Figure 6). Nineteen SNP-trait associations were found significant based on the estimation of reduction in chlorophyll content using a threshold value of p of -log_10_ > 4 (Table 1). The chromosome with the most SNP associations was 6A, followed by 2B. Chromosomes 2B and 6A are flanking a total of 13 MTAs, with three MTAs detected on chromosome 2B and 10 MTAs on chromosome 6A. Chromosomes 3A, 5B, 6B 6D, 7A, and 7B have single MTA each. A Q-Q plot shows the performance of observed value of ps against the expected value of p for association, indicating no effect of population structure and multiple relatedness in SNP prediction (Figure 7). The *R*^2^ values for the 19 significant SNPs explaining phenotypic variations range between 11 and 16%, suggesting that the SNPs represent genomic regions associated with metribuzin tolerance (Table 1). Genomic regions with *R*^2^ > 10% are considered as major QTLs (Xu et al., 2020). Limited information was found on two SNPs located on chromosome 6A (*wsnp_Ku_c20866_30535489* and *wsnp_Ku_c20866_30535750*; Table 2).

**Table 1:** Significant SNPs identified through GWAS of 145 wheat genotypes in response to application of metribuzin by measurement of the reduction in chlorophyll content.

SNP name	Chromosome	Allele	Position	log_10_(*p*)	*R*^2^ (%)
IAAV7130	2B	A/G	657,792,718	4.17	11.67
BS00079013_51	2B	G/A	660,114,463	4.12	11.52
Tdurum_contig5691_596	2B	C/T	668,570,062	4.12	14.07
Tdurum_contig41906_1385	3A	G/A	562,984,283	4.29	14.71
BobWhite_c23687_200	5B	G/A	292,192,552	4.28	14.68
RAC875_c38494_52	6A	T/C	14,607,029	4.75	16.41
RAC875_rep_c79566_238	6A	A/C	14,627,383	4.44	15.25
BobWhite_rep_c50671_356	6A	A/C	14,627,435	4.47	15.36
BobWhite_rep_c50671_408	6A	A/G	14,627,486	4.53	15.58
wsnp_Ku_c20866_30535489	6A	A/G	16,038,973	4.64	16.00
RAC875_c12835_1335	6A	A/G	16,034,906	4.54	15.62
CAP12_c2701_221	6A	T/C	559,526,507	4.16	14.21
Kukri_c29204_358	6A	T/C	561,452,513	4.18	14.29
Kukri_c14906_220	6A	T/C	561,453,969	4.29	14.71
Tdurum_contig41947_720	6A	A/G	583,814,512	4.21	14.40
Excalibur_c9048_1431	6B	A/C	25,091,875	4.21	14.41
wsnp_Ku_c20866_30535750	6D	-	25,091,824	4.44	15.25
CAP7_c1933_170	7A	C/T	484,083,572	4.15	14.20
Kukri_c32241_1165	7B	C/T	49,584,395	4.24	14.50

*R*^2^—variation explained by QTL compared to total phenotypic variation, value of *p* threshold for significant QTL.

**Table 2:** Candidate genes identified as associated with metribuzin tolerance based on GWAS of chlorophyll content index from 145 wheat genotypes.

SNP name	Gene ID	SNP-Gene distance	Gene function	Gene length and direction (bp)
IAAV7130	TraesCS2B01G463800	1,241	protein dimerization activity	3,704+
BS00079013_51	TraesCS2B01G465200	781	ATP binding, protein kinase activity, protein phosphorylation BP	6,356+
Tdurum_contig5691_596	TraesCS2B01G472100	943	GTP cyclohydrolose 1 type 2 homolog	1,882−
Tdurum_contig41906_1385	TraesCS3A02G320100 TraesCS3A02G320200 TraesCS3A02G320300	109,631 71,807 68,839	Acid phosphatase and hydrolase activity BP metal ion binding Protein binding activity Protein binding activity	4,655+ 4,386− 1,068+
BobWhite_c23687_200	TraesCS5B02G158700	539,357	Oxidoreductase activity, acting on the CH-OH group of donors, NAD or NADP as acceptor BP oxidation–reduction process	7,932+
RAC875_c38494_52	TraesCS6A01G028800	3,210	Serine-type endopeptidase activity BP proteolysis	3,686−
BobWhite_rep_c50671_408	TraesCS6A02G028700	23,460	Manganese ion binding activity and nutrient reservoir activity	1,039−
wsnp_Ku_c20866_30535489	No information	–	–	–
BobWhite_rep_c50671_356	TraesCS6A02G028500	143,007	Protein binding activity	1,251+
RAC875_rep_c79566_238 RAC875_c12835_1335	TraesCSU01G079600	8,480	Protein binding activity	15,592+
Tdurum_contig41947_720	TraesCS6A02G353700 TraesCS6A02G354300	1,612,526 1,972,312	Chlorophyllide a oxygenase activity; oxidoreductase activity BP oxidation–reduction process Catalytic activity D-lactate dehydrogenase BP oxidation–reduction process	2,268+ 11,545+
Kukri_c29204_358	TraesCS6A02G328100	882	Embryo sac development arrest 12	3,593+
Kukri_c14906_220	TraesCS6A01G328000	1,733	P-loop containing nucleoside triphosphate hydrolases superfamily protein	1,054+
CAP12_c2701_221	TraesCS6A01G326200	281	Cytochrome b5-like Heme/Steroid binding site, cytochrome b5 reductase (Flavoprotein pyridine nucleotide cytochrome reductase). BP nitric oxide biosynthetic process; and nitrate assimilation oxidoreductase activity	3,385+
Excalibur_c9048_1431	TraesCS6B01G040400	14,129	Protein binding activity	16,024−
wsnp_Ku_c20866_30535750	No information	–	–	–
CAP7_c1933_170	TraesCS7A02G331000	109,918	Cysteine-type peptidase activity (Acetyl-coenzyme A carboxylase carboxyl transferase subunit beta, chloroplastic) BP proteolysis	4,46−
Kukri_c32241_1165	TraesCS7B02G049600	27,098	Signal response regulator, CheY-like superfamily and phosphorelay signal transduction system	219−

−/+ indicates the direction (downstream/upstream) in the genome; bp, represents base pairs.

### Candidate Genes for Metribuzin Tolerance

Eight candidate genes were identified within proximity of the flanked SNP markers, while 11 genes were identified within 2 Mbp distance to flanked SNPs (Table 2). *TraesCS6A01G326200*, located on chromosome 6A, is related to cytochrome P450 pathways that are involved in herbicides stress response in many crops, including wheat. *TraesCS6A02G353700*, located on chromosome 6A, is involved in chlorophyll biosynthesis, and *TraesCS7A02G331000*, located on chromosome 7A, is associated in stress-related responses. *TraesCS2B01G465200*, located on chromosome 2B, involved transmembrane transporter activities relating to xenobiotic detoxification. *TraesCS6A01G028800*, located on chromosome 6A, is associated with many functions in plants, including stress-related responses. Other 14 genes identified from this study are related to protein binding, protein dimerization activities, ion binding, and oxidation-reductive activities which might be related to plant stress metabolism and could provide insight into metribuzin research in the future.

### Validation Result

In this study, five SNPs identified through GWAS were validated using the closest SSR markers to the flanked SNPs. SSR primer sequences (Table 3) were retrieved from the Grain Genes database and used to amplify the sequences of 30 tolerant and 30 susceptible genotypes identified from the phenotypic screening experiment. Polymorphism was observed in only two markers (*wms193* and *barc1036*) validated (Table 3). The amplified product from the gel electrophoresis with “aa” and “AA” alleles corresponds with the susceptible and tolerant genotypes, respectively, as observed during the phenotypic screening experiment (Supplementary Figure S2), suggesting that the primers can be used to differentiate wheat genotypes response to metribuzin application. The mean phenotypic performance of the 30 tolerant and 30 susceptible genotypes was also compared using a *t*-test (*α* = 0.05). The result shows that there is a highly significant difference (value of *p* < 0.001) between the tolerant and the susceptible genotypes confirming the outcomes observed with electrophoresis.

**Table 3:** SSR primers used for validation of 30 top tolerant and 30 most susceptible wheat genotypes.

SSR primers	Position	SSR distance to SNP	Forward and reverse sequence	Size	Annealing temperature
BARC1136-F	567,464,756	4,338,538	CGA GTT TTG CAC AGG ACA ACC AAT A-F	313	50
BARC1136-R	567,464,756	4,338,538	ATG CCA GTT TCT TTC TAG ACA TCT C-R	313	50
BARC113-F	552,302,942	10,823,045	GCGCACAACAACGGACACTTAACAATT-F	234	50
BARC113-R	552,302,942	10,823,045	GGGACTCATTTAGCTTCTACTCGCCATTA-R	234	50
**WMS193-F**	**457,662,948**	**37,250,805**	**CTTTGTGCACCTCTCTCTCC-F**	**216**	**60**
**WMS193-R**	**457,662,948**	**37,250,805**	**AATTGTGTTGATGATTTGGGG-R**	**216**	**60**
**BARC1036-F**	**471,685,345**	**51,273,416**	**CAC CGC AAA AAG ACT TAC AT-F**	**166**	**52**
**BARC1036-R**	**471,685,345**	**51,273,416**	**TGA TGC GTG AGT AAT TCT TTG TAG-R**	**166**	**52**
WMC220-F	633,876,856	2,672,579	GTTTCGAGCGAGGGAGAGT-F	128	60
WMC220-R	633,876,856	2,672,579	GCGTCATTTCCACAAACACC-R	128	60

The ones highlighted in bold are the primers that show polymorphism during electrophoresis.

## Discussion

The current study represents the first report on GWAS that utilized a panel of wheat cultivars with a wide range of genetic variations to identify genomic regions controlling metribuzin tolerance in wheat when used as a pre-emergent. In this investigation, five chromosomes identified through GWAS had been reported to harbor QTLs associated with herbicide tolerance (mostly metribuzin) in wheat by previous investigations. These include chromosomes 5B (Shi et al., 2020) and 2A, 6A, 7A, and 7B (Bhoite et al., 2018; Xu et al., 2020). QTLs that harbor genes associated with photosynthesis are potential genomic regions controlling metribuzin tolerance in wheat (Bhoite et al., 2018; Xu et al., 2020). The QTLs identified in this investigation explained between 11 and 16% of the phenotypic variation and are considered major QTLs. Studies by Shi et al. (2020) also reported the percent of phenotypic variance explained (PVE) ranged from 11.3 to 27.6 where metribuzin was used as a post-emergent. Therefore, these QTLs represent major genomic regions controlling metribuzin tolerance in wheat. For example, in this current study, 10 MTAs are located on chromosome 6A alone. These MTAs are distributed in three blocks: the first block ranges from 14.60 to 14.62 Mbps containing 4 MTAs, two MTAs in close vicinity of 16.03Mbp, and 4 MTAs 55.95–58.38 Mbps.

Therefore, the SNPs found on 6A could be belonging to 3 haplotype blocks stacked on the same chromosome. Xu et al. (2020) reported one major QTL located on the same chromosome. Hence, chromosome 6A may harbor some major genomic regions for metribuzin tolerance in wheat.

Metribuzin tolerance is a quantitative trait involving the contribution of many loci (Bhoite et al., 2019). This suggests that tolerance to metribuzin may be achieved by contribution from different pathways related to PSII, such as activation of defense mechanism in response to harmful foreign material. QTLs identified through the GWAS approach represent a diverse gene pool because of the immense genetic diversity across the panel of genotypes used and could offer a better insight into the underlying genomic region controlling a quantitative trait of interest such as metribuzin tolerance. The various gene annotations within these loci are critical in understanding the functions of the genomic regions and how they can facilitate the genetics of metribuzin in wheat. The current investigation led to identifying 19 genomic regions, harboring genes with known and unknown functions associated with herbicide tolerance pathways. Pathways containing genes that encode proteins, such as the cytochrome P450 superfamilies, are involved in xenobiotic detoxification in many field crops. Likewise, genes encoding ATP binding cassette (ABC) transporters proteins were reported to perform the function of detoxification in plants. Five of the 19 genomic regions identified in this study were associated with pathways relevant to metribuzin tolerance in wheat (Table 3) as reported by previous investigations. These QTLs are distributed across five wheat chromosomes, with candidate genes having annotations across different pathways related to herbicides, stress, and photosynthesis, including genes with unknown functions. Supplementary Figure S3 shows the distribution of different pathways identified in this study.

### Candidate Genes With Annotations Related to Xenobiotic Detoxification

Xenobiotic detoxification of toxic materials, either by modifying the chemical substrate or isolating the chemical from metabolic pathways, is one mechanism that plants adapt to control herbicide stress and develop selectivity (Siminszky, 2006; Xu et al., 2015; Del Buono et al., 2020). This mechanism has been characterized in many field crops, including wheat (Xu et al., 2020). A widely reported herbicide detoxification pathway in plants is the cytochrome P450 (CYP; Siminszky, 2006; Xu et al., 2015, 2020; Bhoite et al., 2019; Nguyen and Dang, 2021). Recently, Bhoite et al. (2019) and Xu et al. (2020) reported CYP superfamilies as one of the major pathways involved in detoxifying metribuzin in wheat and suggested that this superfamily is the pathway implicated most with metribuzin detoxification. Our investigation also revealed that a candidate gene located on chromosome 6A, *TraesCS6A01G326200*, is involved in cytochrome P450 activities. These results confirm the contribution of this genomic region in herbicide resistance. Moreover, the CYP pathway has been reported to be involved in metabolic detoxification of herbicide in weeds such as black grass (Bhoite et al., 2019) and attributed as the source of herbicide resistance mechanism of *Lolium rigidum* (Xu et al., 2020). Identifying this gene in this study resulted from the damage inflicted by metribuzin on the PSII through oxidative stress and suggests that this genomic region may be involved in detoxifying metribuzin molecules. Future research on this topic could focus on studying gene expression through transcriptome analysis to pinpoint the regions associated with CYP in wheat accurately.

Another candidate gene located on chromosome 2B, *TraesCS2B01G465200*, encoding ATP binding cassettes (ABC), has been reported to carry out various functions in living organisms, including xenobiotic detoxification (Davidson et al., 2008; Bhati et al., 2015; Lara et al., 2015). In plants, ABC protein’s primary function is to detoxify harmful materials such as herbicides (Chauhan et al., 2009; Lane et al., 2016). Many ABC proteins have been used to characterize various traits of agricultural importance, including herbicide tolerance (Ezzaldin et al., 1985; He et al., 2018; Do et al., 2021). For example, overexpressed *AtPgpl* protein from the ABC family was utilized by Lloyd et al. (2003) to facilitate a multi-herbicide resistance in *A. thaliana*. A similar approach was also used to confer paraquat tolerance in *A. thaliana* (Xi et al., 2012). In wheat, ABC proteins extracted from seedlings display differential expression for glutathione-mediated detoxification and indicates potential for stress tolerance (Lim et al., 2003; Bhati et al., 2015). Different reports have implicated ABC transporters as metribuzin detoxification pathways in wheat. For instance, Bhoite et al. (2018) reported the involvement of ABC transporters in metribuzin tolerance in wheat and identified genomic regions linked to this superfamily. Xu et al. (2020) also identified genomic regions (QTLs) using bi-parental populations and indicated ABC transporters as a metribuzin detoxification pathway. Current and previous studies indicated that targeted breeding focusing on the genes related to xenobiotic detoxification for developing metribuzin tolerant cultivars would be an essential improvement in dryland farming systems like Western Australia, which depends mainly on herbicides for weed management in wheat paddocks.

### Candidate Gene With Annotation Related to Plant Stress Responses

Stress in plants creates hormonal imbalances, leading to distorted metabolic function and interfering with plant growth and development. In response to stress, plants often adjust various physiological functions to accommodate or compensate for the affected hormone and adapt to harsh environmental conditions (An et al., 2019). A candidate gene, *TraesCS6A01G353700*, located on chromosome 6A, codes for Chlorophyllide *a* oxygenase (CAO), which plays a critical role in stress-related responses. CAO is the enzyme responsible for catalyzing chlorophyll biosynthesis through oxidation of methyl group from chlorophyll *a* to chlorophyll *b*. Hence, CAO is necessary for chlorophyll synthesis in green plants (Yang et al., 2016). The increased chlorophyll content is linked to higher photosynthetic efficiency in crops (Yang et al., 2016), whereas a decrease in chlorophyll content would decrease photosynthetic ability leading to leaf senescence (Pilcher et al., 2017). Herbicides interfere with photosynthesis by decreasing the synthesis of green pigments in chloroplast and eventually reducing chlorophyll content (Yang et al., 2016; Sharma et al., 2020). This suggests that oxidative stress caused by metribuzin application can cause similar effects of chlorophyll degradation. Hence, genotypes with CAO would be expected to have the stay-green phenotype indicative of tolerance to metribuzin. For example, Biswal et al. (2012) used an overexpressed CAO enzyme to improve the photosynthetic efficiency of tobacco plants, thereby improving their potential to withstand prolonged stress. More importantly, a recent transcriptome analysis by Bhoite et al. (2021) implicated chlorophyll *a* and *b* as contributors to metribuzin tolerance in wheat, consistent with the current findings.

### Candidate Genes With Annotations Related to Photosynthesis

Two candidate genes (*TraesCS7A02G331000* and *TraesCS6A01G028800*) identified from this study with functional annotations related to serine, and cysteine-type peptidase activities are known to be involved in defense against pest and pathogen, and in the regulation of plant transition from development through senescing (Jamal et al., 2012; Clemente et al., 2019). As mentioned earlier, herbicide application affects the functioning of the PSII and results in ROS production, leading to degradation of the chlorophyll content and eventual leaf senescing. The endopeptidase role in the PSII reaction center is linked to the D1 protein, consisting predominantly of serine-type endopeptidase (Misra et al., 1991). Genomic regions with functional annotations related to photosynthesis either through control of chlorophyll degradation or control of the activity of the PSII presents excellent potentials for herbicide tolerance in plants. Zhang et al. (2007) showed that leaf senescing in cucumber is directly correlated to increased activity of endopeptidase activities, while peptidase activities are influenced by the reduction in leaf chlorophyll content, suggesting that endopeptidases are a major part of leaf senescing. Pilcher et al. (2017) studied gene expression of susceptible and tolerant wheat cultivars in response to metribuzin application and found out that highly regulated genes under the effects of metribuzin had six times higher senescing associated proteins in tolerant lines compared to susceptible lines. Therefore, during herbicide stress, the underlying factor leading to leaf senescing has to do with the prevention of peptidase activity which is critical for transferring and converting light into energy in the PSII. Hence, a photosynthetic inhibition of the PSII with herbicides such as metribuzin can influence endopeptidase activities, leading to disruption of electron transfer and chlorophyll degradation, as observed in this current study. This outcome is consistent with Bhoite et al. (2018) that proteins associated with the regulation of leaf senescing were found responsible for metribuzin tolerance in wheat. Even though endopeptidase activities and functions are complex and not well understood, and not all endopeptidase proteins are involved in leaf senescence, serine-type endopeptidase is involved with leaf senescing and photosynthesis (Zhang et al., 2007).

### Other Candidate Genes Identified

Other candidate genes identified in this study with annotations related to protein dimerization and binding activities (*Traes CS2B01G463800, Traes CS6A02G028500, Traes CSU01G079600, Traes CS3A02G320200, Traes CS3A02G320300*, and *Traes CS6B01G040400*) and metal ion binding activities (*Traes CS6A02G028700* and *Traes CS3A02G32020*) are not implicated in herbicide or stress-related activities yet. However, they could indirectly be involved in metabolic activities that impact the activities of the genomic regions responsible for metribuzin tolerance (Xu et al., 2020). For example, in wheat, the mutated *NAC* protein binding domain required for protein dimerization has shown positive regulation of leaf senescing (Harrington et al., 2019). Some genes identified from this investigation had annotation of unknown functions, while others had limited information available or unrelated to herbicide tolerance mechanism and, therefore, offer limited potentials.

The approach used in this investigation to identify genomic regions responsible for metribuzin tolerance through GWAS by measuring the relative chlorophyll content index has proven to be very effective in identifying candidate genes for metribuzin tolerance in wheat. However, use of only one parameter for assessing the effect of metribuzin herbicide may limit the number of potential metabolic pathways that can be identified. This, however, did not reduce the validity of the candidate genes identified, only signals that future research may focus on investigating GWAS of metribuzin tolerance in wheat by assessing both the reduction in leaf chlorophyll content through SPAD assessment and leaf damage assessment through visual scoring as done by other investigators such as Bhoite et al. (2019) and Xu et al. (2020).

## Data Availability Statement

The raw data supporting the conclusions of this article will be made available by the authors, without undue reservation.

## Author Contributions

BK, MM, and GY conceived the ideas and designed the experiment. BK conducted the experiment and subsequent analysis with help from MM and prepared the draft. HL collected the materials and conducted the SNP genotyping. MM performs the population structure, LD decay, and GWAS analysis in relevant software. MM, HL, and GY commented and revised the manuscript. All authors contributed to the article and approved the submitted version.

## Funding

The research was partially supported by the Global Innovation Linkage Program (GIL53853) from the Australian Department of Industry, Science, Energy and Resources.

## Conflict of Interest

The authors declare that the research was conducted in the absence of any commercial or financial relationships that could be construed as a potential conflict of interest.

## Publisher’s Note

All claims expressed in this article are solely those of the authors and do not necessarily represent those of their affiliated organizations, or those of the publisher, the editors and the reviewers. Any product that may be evaluated in this article, or claim that may be made by its manufacturer, is not guaranteed or endorsed by the publisher.

## Footnotes

^1^ https://wheat.pw.usda.gov/cgi-bin/GG3/browse.cgi?class=marker

^2^ https://plants.ensembl.org/Triticum_aestivum/Info/Index

^3^ https://plants.ensembl.org/Triticum_aestivum/Info/Index

^4^ https://www.ebi.ac.uk/interpro/

## Abbreviations

SNP: single-nucleotide polymorphism
QTL: quantitative trait loci
GWAS: genome-wide association studies
CCI: chlorophyll content index
PSII: photosystem II
ROS: reactive oxygen species
SSR: simple sequence repeats
MTA: marker-trait association
